# T-cell dysfunction and death in sepsis: a mechanistic review

**DOI:** 10.3389/fimmu.2026.1857308

**Published:** 2026-09-28

**Authors:** Shiyao Ge, Hui Wang, Qixiang Shao

**Affiliations:** 1 Department of Immunology, School of Clinical Medicine and Laboratory Medicine, Jiangsu University, Zhenjiang, Jiangsu, China; 2 Key Laboratory of Novel Technologies for Precision Cancer Diagnosis and Treatment of Huai’an City, Affiliated Hospital of Kangda College, Nanjing Medical University, Lianshui, Jiangsu, China; 3 Institute of Medical Genetics and Reproductive Immunity, The Digestive and Reproductive System Cancers Precise Prevention Engineering Research Center of Jiangsu Province, School of Medical Science and Laboratory Medicine, Jiangsu College of Nursing, Huai’an, Jiangsu, China

**Keywords:** apoptosis, autophagy, ferroptosis, immune checkpoints, immunoparalysis, pyroptosis, sepsis, T cells

## Abstract

Sepsis is a life-threatening syndrome of multi-organ dysfunction caused by a dysregulated host response to infection. Despite advances in supportive care, the underlying immunopathological mechanisms remain incompletely understood. A hallmark of sepsis is immunoparalysis, characterized by an early hyperinflammatory phase followed by sustained immunosuppression, predisposing patients to secondary infections and adverse outcomes. A central feature of this process is profound lymphopenia accompanied by impaired T-cell effector function. This review synthesizes current evidence on T-cell perturbations during sepsis, encompassing numerical depletion, persistent upregulation of inhibitory immune checkpoints, and dysregulated cytokine signaling. We further highlight emerging evidence on four major modes of T-cell loss and dysfunction: apoptosis, autophagy dysfunction, pyroptosis, and ferroptosis, and discuss their integrated impact on immune competence. A deeper understanding of the mechanisms underlying T-cell death may facilitate the development of targeted immunomodulatory strategies and adjunctive therapies for sepsis, an area where effective interventions remain limited.

## 1 Introduction

According to the Third International Consensus Definitions for Sepsis and Septic Shock (Sepsis-3), sepsis is defined as a life-threatening organ dysfunction caused by a dysregulated host response to infection (1). It remains a major global cause of morbidity, mortality, and healthcare expenditure. Recent estimates indicate that sepsis affects nearly 166 million individuals and causes approximately 21 million deaths worldwide each year. In the United States, sepsis-related hospital costs increased from US$28 billion in 2018 to US$52.1 billion in 2021, representing more than 14% of total hospital expenditures, with nearly three-quarters of these costs covered by Medicare and Medicaid (2, 3). Similarly, a national cross-sectional survey of 44 intensive care units in China reported that sepsis accounted for 20.6% of ICU admissions and was associated with a 90-day mortality rate of 35.5% (4). These data collectively underscore the persistent clinical and economic burden of sepsis and the urgent need for improved diagnostic and therapeutic strategies.

Although the pathogenesis of sepsis is incompletely understood, profound dysregulation of immune homeostasis is central to sepsis-induced organ dysfunction. In the early phase, sepsis frequently manifests as a cytokine release syndrome driven by innate or adaptive immune responses to bacterial, viral, or fungal pathogens. Pathogen-associated molecular patterns (PAMPs), including lipopolysaccharide (LPS), lipoteichoic acid, and flagellin, together with damage-associated molecular patterns (DAMPs) such as high-mobility group box 1 (HMGB1), self-DNA, and self-RNA, are recognized by pattern-recognition receptors (PRRs)—including Toll-like receptors (TLRs), C-type lectin receptors, NOD-like receptors (NLRs), and AIM2-like receptors—on macrophages, dendritic cells (DCs), neutrophils, and epithelial cells, thereby triggering robust cellular activation (5, 6). Activated cells release pro-inflammatory cytokines, including interleukin-1 (IL-1), IL-6, IL-18, interferon-γ (IFN-γ), and tumor necrosis factor-α (TNF-α), recruiting additional immune cells and driving excessive myelopoiesis and expansion of myeloid-derived suppressor cells (MDSCs). Neutrophils release granule proteins and form neutrophil extracellular traps (NETs), which facilitate pathogen clearance but can also damage host tissues, particularly the vasculature and lungs (6).

In parallel, activated DCs and macrophages internalize and process pathogen-derived antigens via phagocytosis, whereas B cells capture antigens through B-cell receptor (BCR)-mediated endocytosis and function as antigen-presenting cells (APCs). During sepsis, however, B-cell antigen-presenting capacity is impaired, as evidenced by reduced HLA-DR expression (7), while both B cells and CD4^+^ T cells undergo progressive apoptotic depletion (8). These antigen-presentation processes normally trigger T-lymphocyte activation and differentiation into distinct effector lineages, including T helper subsets (Th1, Th2, Th17, Th9, and Th22) and cytotoxic T-cell populations (Tc1, Tc2, and Tc17).

The initial hyperinflammatory response is rapidly followed by compensatory immunosuppressive mechanisms, including expansion of negative regulatory immune cells such as MDSCs and regulatory T cells (Tregs), increased production of anti-inflammatory cytokines such as IL-10 and transforming growth factor-β (TGF-β), and upregulation of inhibitory immune checkpoints, including programmed cell death protein 1 (PD-1), programmed cell death-ligand 1/2 (PD-L1/PD-L2), and cytotoxic T-lymphocyte-associated protein 4 (CTLA-4). Concurrently, APCs downregulate MHC class II and co-stimulatory molecules (HLA-DR and CD28), further impairing T-cell activation. These alterations ultimately lead to T-cell exhaustion and extensive cell death across multiple immune populations, including T cells, B cells, natural killer (NK) cells, and DCs (9–12). Excessive immunoregulatory responses lead to a profound immunosuppressive state known as immunoparalysis, which compromises the host’s ability to eradicate the primary infection. Consequently, patients develop profound susceptibility to secondary nosocomial infections, characterized by opportunistic bacterial and fungal infections as well as frequent reactivation of latent viruses such as cytomegalovirus and herpes simplex virus (13–15). This prolonged immunosuppressive phase is now widely recognized as a major contributor to the high mortality observed in septic patients (16).

The dynamic progression of T-cell death and dysfunction throughout the stages of sepsis is schematically illustrated in **Figure 1**. In the early hyperinflammatory phase (**Figure 1**, left), PAMPs and DAMPs drive NET formation, pyroptosis, and ferroptosis priming in CD4^+^ T cells. In the mid metabolic crisis phase (**Figure 1**, middle), mTOR activation, mitochondrial dysfunction, and checkpoint accumulation predominate. In the late immunosuppressive phase (**Figure 1**, right), multiple death pathways converge with expanded Treg-mediated suppression, culminating in lymphopenia and immunoparalysis.

**Figure 1:**
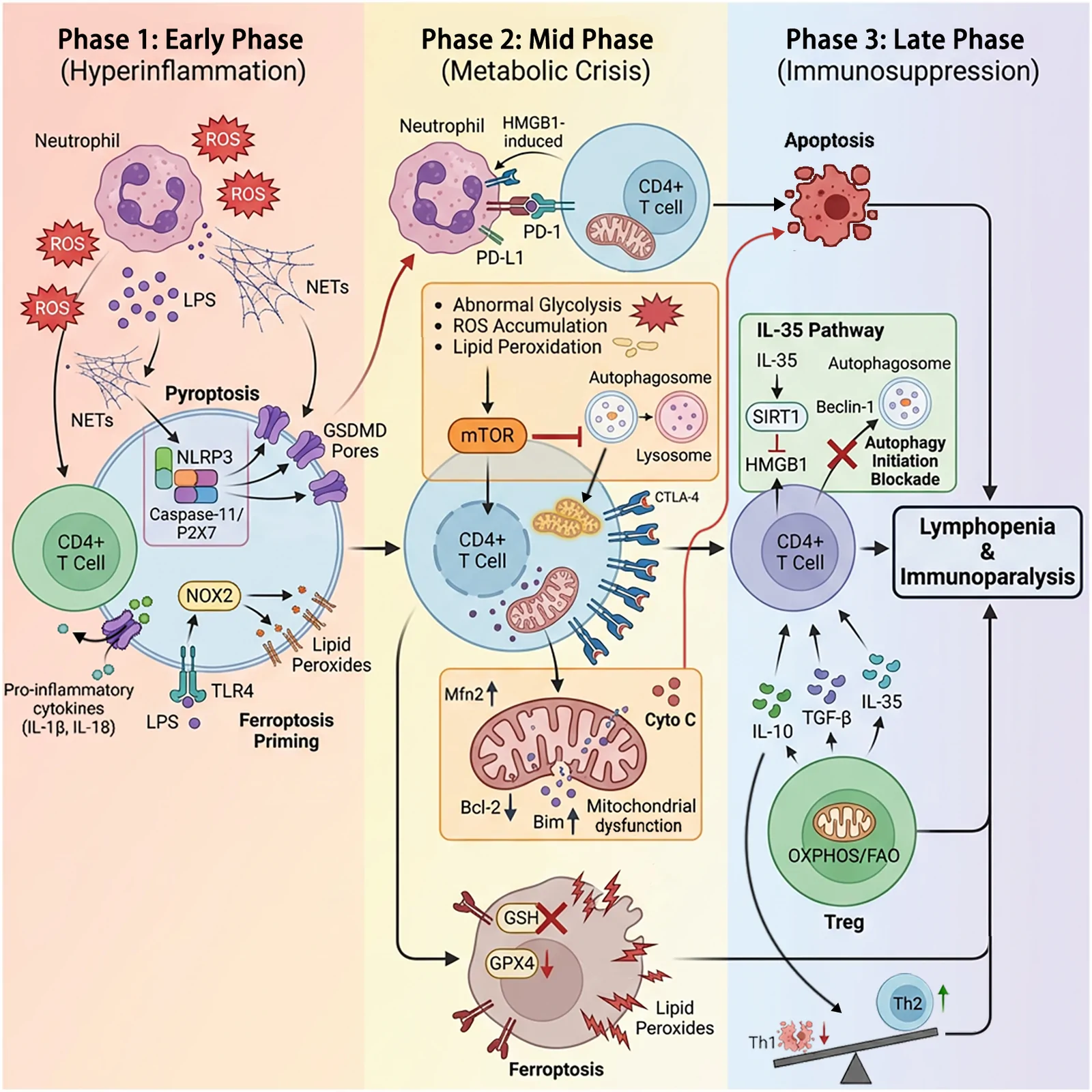
Schematic illustration of the dynamic mechanisms underlying T-cell death and dysfunction across the progressive phases of sepsis. Sepsis progression is divided into three sequential phases. In the early hyperinflammatory phase, PAMPs (e.g., LPS) and DAMPs activate neutrophils and macrophages, leading to ROS generation, NET formation, and release of pro-inflammatory cytokines that trigger pyroptosis (via NLRP3/caspase-11/GSDMD) and ferroptosis priming (via NOX2 and lipid peroxidation) in CD4⁺ T cells. In the mid metabolic crisis phase, abnormal glycolysis, ROS accumulation, and lipid peroxidation activate mTOR signaling, which impairs autophagy, promotes CTLA-4 accumulation, and induces mitochondrial dysfunction (Mfn2 upregulation, Bcl-2 downregulation, and Bim upregulation), while HMGB1–PD-L1 interactions further drive T-cell apoptosis. In the late immunosuppressive phase, persistent dysregulation leads to multiple death pathways, including apoptosis, defective autophagy (via IL-35/SIRT1–mediated Beclin-1 inhibition), and ferroptosis (GSH/GPX4 depletion). Concurrently, expanded Tregs secrete IL-10, TGF-β, and IL-35, suppress effector responses, and promote a Th1-to-Th2 shift, ultimately resulting in lymphopenia and immunoparalysis.

In this review, we summarize current understanding of T-cell dysfunction in sepsis, focusing on numerical depletion, inhibitory receptor upregulation, and cytokine dysregulation. We further highlight recent advances in understanding distinct modes of T-cell death, including apoptosis, autophagy dysregulation, pyroptosis, and ferroptosis, and discuss their implications for the development of novel immunotherapeutic strategies.

## 2 Alterations in T-cell quantity and function during sepsis

T cells are central orchestrators of adaptive immunity. Upon antigen recognition presented by APCs, naive T cells become activated, undergo cytokine-dependent clonal expansion, and differentiate into effector subsets that mediate pathogen clearance. In sepsis, circulating and tissue-resident T cells undergo profound dysregulation that can be characterized by four major features: (1) numerical depletion and subset imbalance; (2) persistent upregulation of inhibitory immune-checkpoint molecules that drive functional exhaustion; (3) impaired effector functions, including reduced proliferative capacity and cytokine production; and (4) disturbed cytokine networks. The following subsections address these dimensions in turn, integrating published clinical, experimental, and single-cell transcriptomic evidence to provide a unified view of T-cell remodeling and death during sepsis.

### 2.1 T-cell numerical depletion and subset imbalance

Clinical studies consistently show marked reductions in absolute numbers of CD3^+^/HLA-DR^+^ lymphocytes in patients with sepsis, accompanied by a decreased CD4^+^/CD8^+^ ratio (17). Such lymphocyte depletion is strongly associated with a higher risk of secondary infection and mortality (18, 19). Persistent lymphopenia is therefore increasingly recognized as a biomarker of sustained immunosuppression in septic patients. Published single-cell analyses of circulating immune cells further indicate that sepsis rapidly remodels T-cell states, with exhaustion-related transcriptional signatures that are most pronounced in fatal disease (20). These findings indicate extensive immune remodeling that may contribute substantially to disease progression.

Importantly, sepsis affects T-cell subsets differentially. Trauma-associated sepsis is frequently accompanied by a reduced CD4^+^/CD8^+^ T-cell ratio that correlates closely with disease severity (21). CD8^+^ T cells also exhibit substantial functional impairment, including decreased antigen sensitivity, attenuated proliferative capacity, and reduced production of effector cytokines. Even regenerated CD8^+^ T cells often display compromised functionality, with diminished IFN-γ and TNF-α secretion that ultimately impairs pathogen clearance (22). CD4^+^ T-cell subsets are likewise profoundly altered: post-injury sepsis is associated with marked expansion of Tregs, an immunosuppressive subset of CD4^+^ T cells, while Th1, Th2, and Th17 subpopulations are significantly reduced (23, 24). Several studies have reported a shift toward Th2-dominant responses accompanied by suppression of Th1 immunity (23). Moreover, a reduced Th17/Treg ratio has been linked to long-term mortality in patients with post-traumatic sepsis (24). Expanded Tregs contribute to immunosuppression through multiple mechanisms, including secretion of anti-inflammatory cytokines (TGF-β and IL-10), upregulation of inhibitory immune-checkpoint molecules (PD-1, TIM-3, TIGIT), induction of effector T-cell death, and metabolic reprogramming that shifts energy utilization from glycolysis toward oxidative phosphorylation (OXPHOS) (25).

Importantly, Treg-mediated suppression is not restricted to a single effector subset but constitutes a broader regulatory network that influences the survival, proliferation, and functional differentiation of both CD4^+^ and CD8^+^ T cells. Through the secretion of inhibitory cytokines (IL-10 and TGF-β) and competition for critical costimulatory and survival signals via CTLA-4 and high-affinity CD25, persistently expanded Tregs directly constrain the proliferation, effector differentiation, and survival of naive and memory T-cell pools. The coexistence of CD8^+^ T-cell exhaustion, impaired Th1/Th17 immunity, and persistent Treg expansion generates a self-reinforcing immunosuppressive environment that ultimately prevents effective pathogen clearance despite resolution of the initial inflammatory response.

Collectively, these observations indicate that both quantitative loss and compositional imbalance of T-cell subsets are critical drivers of sepsis-associated immunosuppression. Thus, sepsis-induced immunoparalysis should not be regarded merely as numerical T-cell depletion, but rather as the result of disrupted communication and functional imbalance among multiple T-cell compartments. Understanding these inter-subset interactions may provide a more comprehensive framework for the development of immune-restorative therapies.

### 2.2 Immune checkpoint regulation of T-cell dysfunction in sepsis

Building on the numerical and compositional changes described above, a second major dimension of T-cell dysregulation is the persistent upregulation of inhibitory immune-checkpoint molecules that actively drive functional exhaustion. T-cell exhaustion is classically defined as progressive functional decline during persistent antigenic stimulation (26). The sustained upregulation of inhibitory immune-checkpoint molecules, particularly PD-1 and CTLA-4, has been closely linked to lymphopenia, impaired cytokine production and poor clinical outcomes. The PD-1/PD-L1 signaling axis plays a central role in sepsis-associated immune dysfunction (27). Increased PD-1 expression on CD8^+^ T cells correlates strongly with adverse clinical prognosis (28), whereas elevated PD-1 on T cells combined with increased PD-L1 on APCs is associated with defective T-cell proliferation and increased mortality risk in septic patients (29). Preclinical studies have demonstrated that therapeutic blockade of PD-1 or PD-L1 signaling partially restores T-cell function and significantly improves survival in murine sepsis models (30). Furthermore, a phase Ib clinical trial investigating the anti-PD-1 antibody nivolumab (NCT02960854) reported acceptable safety profiles in septic patients, although clinical efficacy remains to be validated in larger trials (31).

In addition to PD-1 and CTLA-4, several other inhibitory receptors, including TIM-3, are upregulated on CD4^+^ T cells during sepsis. These checkpoint molecules are associated with immune dysfunction, and their expression increases both in septic patients and in the murine cecal ligation and puncture (CLP) model. Notably, blockade of these pathways improves survival in experimental bacterial or fungal sepsis, highlighting their translational potential (32, 33). Mechanistically, sustained activation of inhibitory checkpoint signaling suppresses T-cell receptor-mediated activation, reduces cytokine production, and impairs effector functions, thereby contributing to the development of sepsis-induced immunoparalysis. Furthermore, the co-expression of multiple inhibitory receptors may represent a progressive state of T-cell exhaustion rather than a single-pathway defect. Collectively, these findings suggest that inhibitory immune-checkpoint molecules, particularly PD-1, may serve as both prognostic biomarkers and therapeutic targets in sepsis.

### 2.3 Dysregulation of cytokine signaling in septic T cells

The molecular alterations described above ultimately converge on impaired T-cell effector function, most notably the disruption of cytokine production and homeostatic cytokine signaling. Cytokine production by T cells is profoundly disrupted during sepsis and represents a hallmark of sepsis-induced immunosuppression. Accumulating evidence indicates that IFN-γ and IL-7 play critical roles in orchestrating antimicrobial responses. IFN-γ enhances macrophage-mediated bacterial phagocytosis, whereas IL-7, a critical homeostatic cytokine, sustains T-cell survival, promotes homeostatic proliferation, and prevents activation-induced cell death through the upregulation of anti-apoptotic Bcl-2 family members. However, in septic patients, circulating T cells exhibit markedly impaired proliferative capacity and reduced IFN-γ production, and IL-7–mediated homeostatic support is inadequate to restore T-cell competence, contributing to tissue injury and high morbidity and mortality (16, 34).

Given these insights, IL-7 has emerged as a promising immunorestorative candidate. Studies have shown that IL-7 administration reverses sepsis-associated lymphopenia, restores T-cell function, and enhances IFN-γ production, even in the setting of multidrug-resistant bacterial infections (35). Consequently, recombinant human IL-7 and IFN-γ are being investigated as potential therapies for sepsis. In a prospective, randomized, double-blind phase II clinical trial involving 27 patients with septic shock and severe lymphopenia, recombinant human IL-7 significantly restored CD4^+^ and CD8^+^ T-cell counts without exacerbating systemic inflammation (36). Ongoing clinical trials are also evaluating IFN-γ therapy in immunoparalyzed septic patients (37). Although these results are encouraging, large-scale trials are still required to confirm clinical efficacy and optimize dosing strategies.

## 3 Mechanisms of T-cell death in sepsis

Regulated cell death (RCD) programs are not uniformly engaged across T-cell subsets during sepsis. Published experimental and clinical studies indicate that effector and helper subsets are more vulnerable to apoptosis and pyroptosis, whereas Tregs are relatively resistant, in part through higher anti-apoptotic signaling. The phase-specific mechanisms of T-cell death are summarized schematically in **Figure 1**. The following subsections examine each major pathway in turn.

### 3.1 Apoptosis in sepsis-induced T-cell loss

Apoptosis is a tightly regulated, non-inflammatory form of programmed cell death characterized by caspase activation, membrane blebbing, and orderly cellular dismantling. In sepsis, enhanced T-cell apoptosis is a major driver of lymphopenia and immunosuppression. During the early phase of sepsis, a rapid and profound decline in CD4^+^ T-cell counts is frequently observed, which is largely attributed to increased circulating T-cell apoptosis, impaired thymocyte maturation, and reduced thymic output of CD4^+^ T cells (8). The extent of lymphocyte apoptosis correlates with the severity of lymphopenia and mortality in septic shock (8, 16).

Mechanistically, Th1 cells are particularly susceptible to mitochondrial apoptosis, while Th2 cells are relatively preserved, reinforcing a Th2-dominant immunosuppressive environment (38, 39). IL-10 further enhances Fas/FasL-mediated apoptosis of Th1 cells without substantially altering the transcriptional program of Bcl-2 family proteins (40, 41). Murine models of sepsis demonstrate marked expansion of splenic Treg populations during disease progression (39). Metabolic reprogramming in activated Tregs—from oxidative phosphorylation to glycolysis and fatty-acid oxidation (FAO)—confers increased resistance to apoptosis (42). Tregs can also induce apoptosis in effector T cells through TGF-β1-mediated mechanisms (43).

In addition, immune-checkpoint signaling contributes to T-cell apoptosis during sepsis. The PD-1/PD-L1 pathway has been identified as an important mediator of septic T-cell loss. HMGB1 induces PD-L1 expression on neutrophils through TLR2 signaling (44), thereby promoting PD-1/PD-L1-dependent apoptosis of T cells and aggravating lymphopenia (29). Therapeutic blockade of PD-1 signaling using anti-PD-1 antibodies rescues T-cell apoptosis and improves survival in murine models of sepsis (45). Intrinsic mitochondrial apoptotic pathways are also activated in septic T lymphocytes. HMGB1 has been shown to upregulate mitofusin 2 (Mfn2) in CD4^+^ T cells, thereby inducing mitochondrial dysfunction and apoptosis (46). Moreover, CLP-induced sepsis models demonstrate reduced expression of the anti-apoptotic protein Bcl-2 and increased expression of the pro-apoptotic molecule Bim, which collectively promote mitochondrial-dependent T-cell apoptosis (47). Conversely, IL-33 preserves Bcl-2 expression and enhances T-cell survival following CLP challenge, indicating the existence of endogenous protective mechanisms (48). Plasma-derived exosomes from septic patients enriched in miR-7-5p have been shown to downregulate Bad expression, increase Bcl-2 levels, and attenuate LPS-induced T-cell apoptosis *in vitro* (49). These findings highlight the complex regulation of T-cell apoptosis by both pathogenic signals and protective extracellular mediators during septic immune dysregulation.

Collectively, apoptosis preferentially depletes effector and Th1 populations, contributes directly to numerical loss, and amplifies functional imbalance among T-cell subsets. These apoptotic processes are particularly prominent in the mid-to-late phases of sepsis (**Figure 1**, middle and right panels). Therapeutic blockade of PD-1 or enhancement of anti-apoptotic signals (e.g., IL-7, IL-33) has shown benefit in preclinical models.

### 3.2 Dysregulation of autophagy in T cells

Autophagy is a conserved catabolic process that maintains cellular homeostasis by degrading damaged organelles and macromolecules via lysosomes. Under physiological conditions it supports T-cell survival, activation, and cytokine production. During sepsis, however, autophagic flux is impaired in both CD4^+^ and CD8^+^ T cells, as evidenced by reduced LC3-II and ATG7 expression and decreased autophagosome–lysosome fusion (50, 51).

This impairment is driven largely by persistent mTOR activation. Increased mTOR activity constrains autophagy, promotes CTLA-4 accumulation, and increases apoptotic susceptibility (50, 52, 53). Genetic deletion of Atg7 in CD4^+^ and CD8^+^ T cells markedly increases T-cell apoptosis and mortality after CLP (54). Disruption of autophagy also suppresses production of Th1, Th2, and Th17 cytokines (51). Sepsis-associated metabolic disturbances further contribute to sustained mTOR activation, leading to autophagy impairment and immunoparalysis (55).

Cytokine signals modulate the pathway: IL-35 (produced by Tregs) restricts HMGB1 nuclear export via SIRT1-mediated deacetylation, thereby preventing HMGB1–Beclin-1 interaction and inhibiting autophagy in effector CD4^+^ T cells (56, 57). In contrast, IL-36β induces robust autophagy in Tregs, reducing their suppressive activity and enhancing host responses in CLP models (58, 59).

mTOR-driven autophagy impairment is a hallmark of the metabolic crisis phase (**Figure 1**, middle panel). Consequently, defective autophagy lowers the threshold for apoptosis and pyroptosis, amplifies checkpoint-mediated dysfunction, and contributes to sustained immunoparalysis. Pharmacological mTOR inhibition (e.g., rapamycin) can restore autophagic activity and improve outcomes in experimental sepsis (50, 52).

Taken together, these findings highlight autophagy as a crucial protective pathway that supports T-cell survival and function, yet becomes disrupted in sepsis through metabolic, cytokine-mediated, and mTOR-dependent mechanisms.

### 3.3 Pyroptosis in septic T cells

Pyroptosis is a lytic, highly inflammatory form of regulated cell death executed by gasdermin pores following inflammasome activation. In certain infection settings it can support protective T-cell responses (60, 61); however, in sepsis it predominantly drives pathogenic T-cell loss. Circulating mononuclear cells from septic patients show elevated pyroptotic activity, and NLRP3-dependent pyroptosis correlates with lymphopenia, organ dysfunction, and mortality (62, 63).

CD4^+^ T cells are eliminated through the canonical pyroptosis pathway during the immunosuppressive phase of sepsis (64). LPS-driven activation of the caspase-11/P2X7 axis increases pyroptosis in gut mucosal CD4^+^ T cells (65). The mTOR pathway also modulates pyroptosis by negatively regulating PPARγ–Nrf2 signaling or autophagy activity (66).

Neutrophil-derived NETs provide an important upstream trigger. NETs are markedly elevated in septic lymphopenia and correlate with activation of NLRP3, caspase-1, and GSDMD in CD4^+^ T cells (67–69). Although NETs can activate inflammasomes, the precise molecular mechanisms through which neutrophil granule contents induce T-cell pyroptosis remain to be fully elucidated (68).

NET-triggered pyroptosis is initiated early and continues to contribute to T-cell loss in later stages (**Figure 1**, left and right panels). Thus, pyroptosis preferentially affects activated effector populations, links innate (NET) and adaptive immune dysregulation, and amplifies both inflammation and lymphopenia. Inhibition of GSDMD, PAD4-dependent NETosis, or upstream inflammasome components represents a potential therapeutic avenue.

### 3.4 Ferroptosis in septic T cells

Beyond apoptosis, autophagy, and pyroptosis, emerging evidence indicates that ferroptosis also contributes to T-cell dysfunction during sepsis. Ferroptosis is an iron-dependent form of programmed cell death driven by the accumulation of oxidized phospholipids (OxPL) and characterized by impaired glutathione peroxidase 4 (GPX4) activity. In sepsis, profound disturbances in iron metabolism and lipid oxidation create a permissive environment for ferroptosis. Patients with sepsis exhibit distinct alterations in metabolic pathways, including increased reliance on FAO and elevated circulating monounsaturated fatty acids, which potentiate inflammatory responses and tissue injury (55, 70).

Recent analysis of four pediatric sepsis datasets has identified GPX4 as a key biomarker associated with immune dysfunction and T-cell inhibitory molecules in pediatric sepsis (71). Depletion of glutathione (GSH)—an essential cofactor for GPX4—further accelerates lipid peroxidation and ferroptosis (72, 73). Increasing studies have elucidated the crucial effects of ferroptosis on T cells in sepsis. CD4^+^ T cells from patients with severe polytrauma exhibit iron overload, diminished GSH and GPX4 levels, and mitochondrial morphological features consistent with ferroptosis. Notably, ferroptotic depletion of CD4^+^ T cells via the GSH–GPX4 pathway independently predicts the subsequent development of sepsis in this population (74). CD4^+^ T-cell ferroptosis has been implicated in sepsis-associated acute kidney injury, where microbial and metabolic signals converge on T-cell NOX2/ROS pathways. Short-chain fatty acid acetate can attenuate this process (75).

Ferroptosis priming begins in the early phase and becomes more evident as metabolic and redox stress accumulates (**Figure 1**). Ferroptosis therefore contributes an additional, metabolically driven route of T-cell loss that intersects with oxidative stress and mitochondrial dysfunction.

### 3.5 Integrative perspective: crosstalk among death pathways

Although apoptosis, pyroptosis, ferroptosis, and autophagy dysregulation possess distinct execution mechanisms, they do not operate in isolation during sepsis. T cells are exposed to shared upstream stresses—excessive ROS, mitochondrial damage, sustained inflammatory cytokines, and metabolic exhaustion—that simultaneously lower the threshold for multiple death programs (76).

Experimental evidence indicates several points of intersection: mTOR-dependent autophagy impairment sensitizes cells to both apoptosis and pyroptosis; NET-derived signals can trigger pyroptosis while also promoting oxidative stress that favors ferroptosis; and loss of GPX4 activity can amplify lipid-ROS that feed back into mitochondrial apoptotic pathways.

Consequently, the ultimate fate of septic T cells is governed by dynamic crosstalk and compensatory switching among these death modalities, which collectively accelerate progressive lymphocyte depletion, profound exhaustion, and sustained immunoparalysis (as summarized in **Figure 1**).

## 4 Therapeutic strategies targeting T-cell death and immune restoration in sepsis

Given that T-cell death in sepsis arises from an interconnected network of apoptotic, pyroptotic, ferroptotic, and autophagic pathways rather than from any single modality, therapeutic strategies are best approached as multi-layered. Effective intervention requires simultaneous attention to upstream triggers (such as NETs and metabolic stress), core execution pathways, survival and recovery signals, and the durable epigenetic programs that lock cells into an immunosuppressive state. The following subsections address these complementary approaches.

### 4.1 Targeting pyroptosis and NETosis to limit T-cell loss

Pyroptosis and NETosis are closely linked drivers of T-cell depletion in sepsis. NETs release histones, DNA, and granule proteins that can activate the NLRP3 inflammasome and promote GSDMD-dependent pyroptosis in neighboring T cells. Consequently, strategies that interrupt either NET formation or GSDMD pore formation have the potential to protect T cells while also mitigating sepsis-associated coagulopathy.

Several agents targeting the caspase–GSDMD axis have shown benefit in preclinical models. Necrosulfonamide (NSA) inhibits GSDMD oligomerization at Cys191, reduces serum IL-1β, and improves survival (77, 78). Disulfiram, another GSDMD inhibitor, suppresses both pyroptosis and NET release, thereby attenuating multi-organ dysfunction (77, 79). Broad-spectrum caspase inhibition with VX-166 decreases IL-18 release and lung injury (80, 81). Natural compounds such as berberine and tea polyphenol nanoparticles also interfere with caspase-11 or GSDMD activity (78, 82). In addition, dimethyl fumarate (DMF) and 4-octyl itaconate (4-OI) limit caspase-11-mediated pyroptosis and tissue-factor release (83, 84). Heparin, already used clinically, can block cytosolic LPS delivery and caspase-11 activation (85).

In addition to strategies targeting the caspase–GSDMD axis, parallel efforts have focused on NETosis itself. Inhibitors of peptidylarginine deiminase 4 (PAD4) suppress histone citrullination and NET formation, preserving dendritic-cell activation and improving antigen presentation to T cells (86). Mesenchymal stem cell-derived apoptotic vesicles can redirect neutrophils from NETosis toward apoptosis, further reducing organ injury (87). By limiting NET-derived danger signals, these approaches simultaneously protect T cells from pyroptotic death and reduce vascular inflammation.

### 4.2 Promoting T-cell survival and functional recovery

Even when acute cell death is successfully limited, sepsis often leaves a prolonged defect in lymphoid recovery. This reflects a broader impairment of immune reconstitution, in which surviving T cells may remain functionally exhausted or fail to expand normally. Strategies that directly enhance T-cell survival and effector function are therefore essential to immune restoration.

Cytokine therapy remains the most advanced approach. IL-7 and IL-15 upregulate Bcl-2, inhibit apoptosis, and restore T-cell numbers and function. In preclinical models, IL-7 increases CD4^+^ T-cell counts by approximately 30% and improves bacterial clearance (88, 89). A phase II trial demonstrated that recombinant human IL-7 safely expands both CD4^+^ and CD8^+^ T cells in patients with septic shock and severe lymphopenia (36). In clinical settings, granulocyte-macrophage colony-stimulating factor (GM-CSF) therapy significantly reduces MDSCs and Foxp3^+^ Tregs, enhances CD4^+^ T-cell function, and improves patient survival (90, 91).

In addition to direct cytokine administration, advanced delivery platforms have been developed to achieve more precise immune restoration. Lipid nanoparticles co-delivering ApoA1 and IL-4 that target hematopoietic organs can alleviate immune paralysis in both murine and ex-vivo human models (88). IL-7 further enhances CD8^+^ T-cell cytotoxicity through CD127 and STAT5 signaling (89, 92). Emerging work on NUFIP1-mediated ribophagy suggests that quality-control pathways intersecting with cGAS–STING signaling may also be leveraged to limit PANoptosis-like death of CD4^+^ T cells (93). This finding highlights a potential regulatory mechanism linking cellular quality-control pathways with T-cell fate determination and provides a theoretical foundation for developing precision strategies targeting immune dysfunction in sepsis.

### 4.3 Rebalancing effector and regulatory T-cell compartments

During the immunosuppressive phase of sepsis, Tregs accumulate in part because they are relatively resistant to apoptosis and continue to suppress effector T-cell responses through IL-10, TGF-β, and checkpoint molecules (94). Complete depletion of Tregs risks uncontrolled inflammation; therefore, the therapeutic goal is selective rebalancing rather than their elimination.

Preclinical studies have explored several concrete approaches. Carefully timed or partial depletion with anti-CD25 antibodies can reduce Treg numbers, improve bacterial clearance, and enhance survival in secondary-infection models after sepsis (95, 96). Blockade of co-inhibitory receptors enriched on Tregs, such as CTLA-4, has been shown to reverse late-phase immunosuppression; low-dose anti-CTLA-4 improves survival in experimental sepsis, whereas higher doses may be detrimental (97). Genetic or pharmacologic interference with the chemokine receptor CCR4, which is highly expressed on Tregs, attenuates their suppressive activity and improves host defense and survival after CLP (98). In addition, modulation of TNFR2 signaling on Tregs is under investigation as a means to selectively limit their inhibitory function. Timing remains critical: interventions are likely to be most beneficial after the initial hyperinflammatory phase has subsided but before durable immunoparalysis is established.

### 4.4 Epigenetic regulation of T-cell function

Persistent T-cell exhaustion and Treg-mediated suppression are partly maintained by stable epigenetic imprints, including altered DNA methylation and histone modifications. These durable changes may help explain why functional defects often outlast the resolution of acute inflammation.

Several epigenetic agents have shown activity in preclinical sepsis models. The HDAC1-selective inhibitor mocetinostat reverses CD8^+^ T-cell exhaustion, reduces inhibitory receptor expression, increases circulating CD8^+^ T-cell numbers, and improves survival in a two-hit sepsis model (99). Broader HDAC inhibitors such as trichostatin A (TSA) and suberoylanilide hydroxamic acid (SAHA) attenuate inflammation, limit organ injury, and improve survival after CLP or endotoxemia (100, 101). DNA methyltransferase inhibitors, including decitabine, reduce NF-κB activation, lower inflammatory cytokine levels, and significantly improve survival in CLP-induced severe sepsis (102). Combined HDAC and DNMT inhibition has demonstrated synergistic protective effects in experimental models. Although these agents have not yet been systematically evaluated for septic T-cell reprogramming in patients, the existing preclinical data support the concept that epigenetic remodeling may achieve more durable restoration of immune competence than single-pathway interventions alone.

## 5 Concluding remarks and future perspectives

Sepsis is a complex syndrome driven by a dysregulated host response to infection, progressing from early hyperinflammation to sustained immunosuppression. Among immune cell populations, T cells undergo profound numerical depletion and functional impairment, largely as a consequence of multiple, overlapping forms of programmed cell death.

This review synthesizes emerging insights into four major T-cell death pathways in sepsis: apoptosis, autophagy dysfunction, pyroptosis, and ferroptosis. These mechanisms rarely operate in isolation. Instead, distinct T-cell subsets—including Th1, Th2, Th17, and Tregs—exhibit divergent vulnerabilities influenced by metabolic status, cytokine signaling, and immune checkpoint pathways. Growing evidence also highlights extensive crosstalk among these death modalities. For example, mTOR-dependent suppression of autophagy may potentiate both apoptosis and pyroptosis, whereas metabolic disturbances can predispose T cells to ferroptosis. Additionally, neutrophil-derived NETs can trigger T-cell pyroptosis, thereby linking innate immune activation to adaptive immune cell death.

Published single-cell studies of circulating immune cells in sepsis support a model in which T-cell states are rapidly remodeled and exhaustion-related programs emerge, particularly in severe or fatal disease. These observations are consistent with experimental evidence that T-cell loss reflects the convergence of functional exhaustion and interconnected cell-death pathways. Although murine models have provided fundamental mechanistic insights, translation to human sepsis remains challenging. Key features such as T-cell apoptosis, lymphopenia, checkpoint upregulation, and functional exhaustion are observed in both settings. However, important biological discrepancies must be acknowledged. Mice exhibit higher endotoxin tolerance and divergent leukocyte transcriptional responses compared with humans. Standard experimental models (LPS challenge and CLP) typically use young, genetically homogeneous animals with synchronized insults, whereas human sepsis is highly heterogeneous with respect to age, comorbidities, pathogen, treatment, and timing. Bridging these gaps will require humanized models, organ-on-a-chip platforms, and longitudinal clinical multi-omics validation.

Emerging evidence further suggests that T-cell depletion in sepsis may involve interconnected regulated cell death pathways rather than isolated death modalities. Recent work has implicated NUFIP1-mediated ribophagy in PANoptosis-like death of CD4^+^ T cells via the cGAS–STING pathway (93). Whether a canonical PANoptosome complex is assembled in septic T cells, however, remains to be established. Future single-cell and multi-omics studies are needed to define subset-specific death programs and the molecular interactions among pathways.

A deeper understanding of these interconnected mechanisms will be essential for developing effective immunomodulatory therapies. Because T-cell death in sepsis arises from a networked rather than linear process, therapeutic strategies are most likely to succeed when they address multiple layers simultaneously—limiting upstream triggers such as NETs, interrupting core execution pathways, promoting T-cell survival and functional recovery, rebalancing effector and regulatory compartments, and, where possible, remodeling the epigenetic programs that sustain exhaustion. Single-pathway interventions (e.g., PD-1/PD-L1 blockade, mTOR inhibition, or GPX4 activation) have shown promise, yet compensatory switching among death programs frequently limits their efficacy. Future approaches should therefore aim at the broader cell-death network and its upstream drivers rather than any single modality.

Dysregulation of cell-death signaling and immune homeostasis lies at the core of sepsis pathogenesis. With continued advances in understanding pathway crosstalk and the development of multi-layered targeted agents, modulation of T-cell death and dysfunction is poised to become an important component of precision immunotherapy for sepsis.
